# Perceived determinants of implementation success of the neglected tropical diseases programme in Ghana: a qualitative study among programme officers

**DOI:** 10.1186/s12889-021-12096-7

**Published:** 2021-11-11

**Authors:** Desmond Dzidzornu Otoo, Nana Nimo Appiah-Agyekum, Francis Anderson Adzei

**Affiliations:** Department of Public Administration and Health Services Management, University of Ghana, Accra, Ghana

**Keywords:** Neglected, Endemicity, Co-endemicity, Tropical, Implementation, Determinant

## Abstract

**Background:**

The importance of health policy implementation cannot be overemphasized in contemporary public health. Neglected Tropical Diseases (NTDs) have negatively impacted society, affect quality of life and make the poor societies poorer. Several policies and strategies have been put in place across the world including the neglected tropical diseases programme in Ghana. Though chalked many successes, the programme continues to lag behind in the full attainment of various objectives. Several factors exist that determine how effective a programme is implemented. Identification of these factors on every programme is essential to determine where more programme resources need to be channelled. This study assessed the determinants of successful implementation of the neglected tropical diseases programme in Ghana.

**Methods:**

A qualitative approach with the case study design was employed. Purposive and snowball sampling techniques were used to identify key programme officers at the national, regional and district levels of programme implementation. Eighteen (18) Key informant interviews were conducted at all the three levels of the Ghana Health Service NTDs programme. Data were thematically analysed and presented.

**Results:**

Findings from the study revealed that determinants that influenced the successful implementation of the NTDs programme include donor support, education and training, partnerships, reliability of the health structure, integrative nature of the programme and management commitment. These determining factors cut across the inner settings of the implementing agency and the external environment.

**Conclusion:**

Neglected tropical diseases continuously affect Ghanaians, especially the poor. It is important for both policy makers and implementers to identify the factors that ensure the success of the programme in the Ghanaian context. Though the factors are independently sufficient, they synergistically lead to improved programme implementation. Empowering all units involved (local to national level) and maximizing the enabling factors identified to would improve upon implementation and ensure sustainability.

## Background

Health policy implementation has been a critical issue in the local, national and international discourse and its importance cannot be overemphasized. The policy cycle is deemed incomplete without effective implementation of relevant policies and the success of any programme is determined largely by the effectiveness of its implementation [1]. Health policy and programme implementation is widely viewed as the process by which governments and other stakeholders put strategies embedded in policies in place to effect changes in the health system [2, 3]. While innovative policy and strategy formulation is sufficient to champion an organization to success [4], ensuring that such policies and strategies work to meet set objectives is very crucial.

Globally, implementation of strategies is bedevilled with many challenges. There are reported disparities between policy formulation and implementation among many regions and economies [1]. Despite the efforts of health ministries, agencies and bodies to ensure smooth and effective implementation process, reports indicate that majority of plans are not executed as outlined in various documents and do not entirely meet set objectives. This may be due to known and unknown factors that are not sufficiently controlled. Developed countries and economies tend to have higher success rates in terms of implementation as compared to developing countries [5, 6].

Neglected Tropical Diseases (NTDs) have been conceptualized as a wide group of infectious diseases that thrive in tropical and subtropical regions. It is estimated that about one [1] billion people in the world are affected by one or more NTDs and such predicaments place huge costs on these affected countries every year [7]. Populations that are highly affected most often live in very poor communities with inadequate resources and poor living conditions. Due to the increasing burden of NTDs and the re-emergence of diseases, the World Health Organization (WHO) introduced measures to control, eliminate and eradicate most of the diseases. WHO started working with member states in the year 2013 to intensify efforts against NTDs and in 2016, it adopted a resolution to implement appropriate and specific measures to eliminate many and eradicate at least two of the NTDs by the year 2020. This resolution has been the objective of many WHO member countries who have developed the neglected tropical diseases programme [7, 8].

The Neglected Tropical Diseases Programme (NTDP) of Ghana was introduced in 2006 with a team, and office at the national level. However, implementation wholly depends on the health system from the regional to the district level. As part of the programme, a more recent NTDs master plan (2016–2020) was developed to serve as a guideline to the NTDs programme with the goal to improve on the capacity of the health agencies in order to deliver interventions that will prevent, control, eliminate or eradicate the NTDs by the year 2020. This was designed to cover preventive therapy and case management [9, 10].

Since its introduction, implementation has been fairly successful despite some objectives of the programme not fully met. It is reported that policies and strategies do not inherently fail on their own, they are subjected to complex systems within the policy context [1]. Thus, it is essential to identify these factors within the health system in order to increase effectiveness of the programme. An assessment of the implementation of the programme is needed to identify factors facilitating and hindering the implementation process.

## Methods

### Study design/approach

The study employed a qualitative research approach to study and enquire about implementation across various levels. The qualitative approach was used in this study to enable the researcher to explore and understand implementation as a social phenomenon [11]. Hence, the adoption of the qualitative approach in the study at the three levels of healthcare in Ghana ensured that an in-depth understanding was obtained through a deductive process [12].

A case study design was used to obtain an in-depth understanding of the programme within the context of the Ghana Health Service. It investigates and explores issues through the lens of a deep contextual analysis and dwell on a limited number of events and how they relate [13]. This study focused on a descriptive case study in order to describe the implementation of the neglected tropical diseases programme within a particular context in order to create an understanding of the various determinants of implementation success.

### Study area

The study considered the neglected tropical diseases programme at the Greater Accra and Eastern regional health directorates, Ga West municipality in the Greater Accra Region and the Lower Manya Krobo municipality in the Eastern Region of Ghana. These areas were selected due to convenience, urban, peri-urban and rural balance and co-endemicity of two or more neglected tropical diseases.

**Fig. 1:**
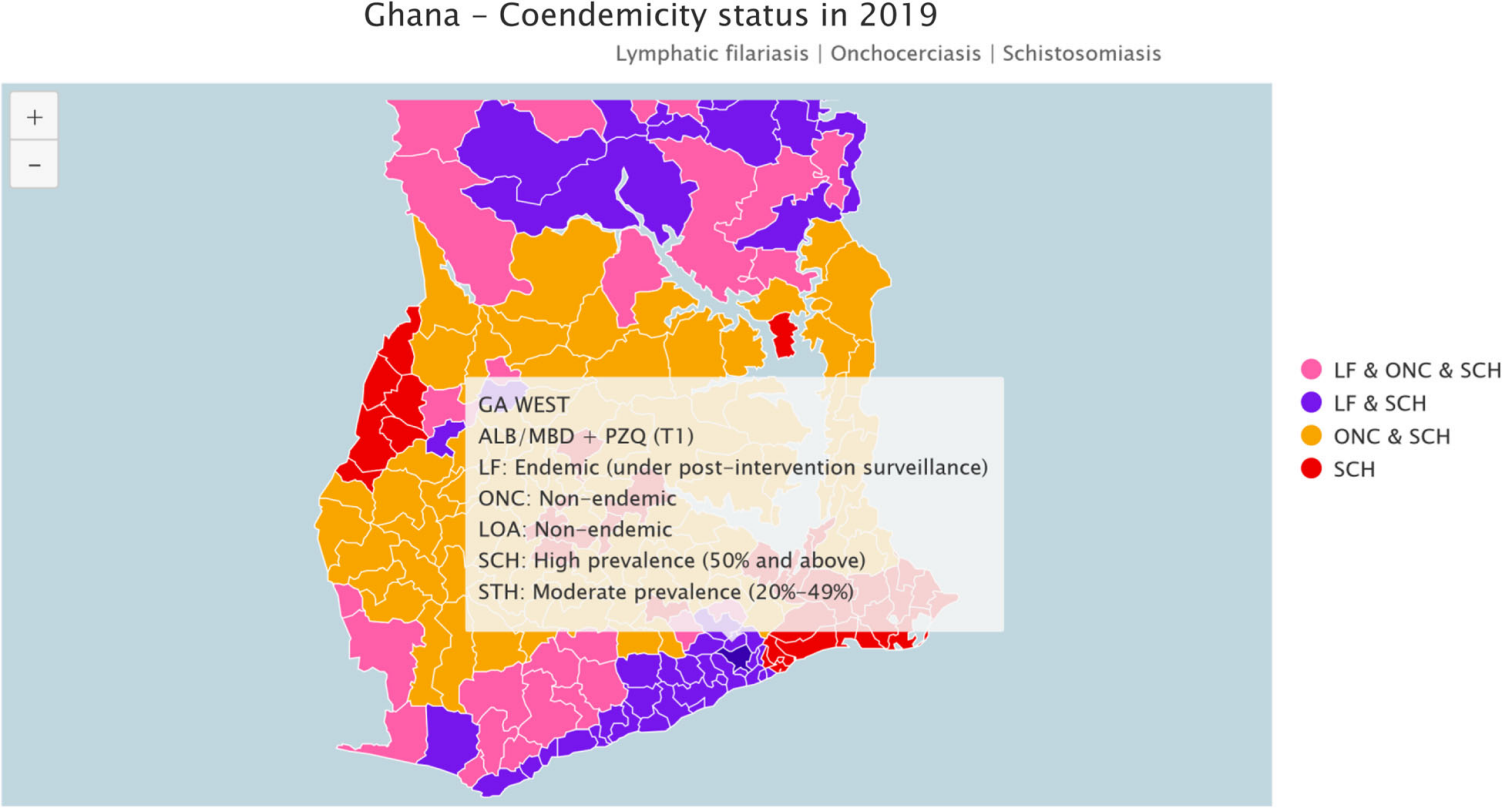
Map showing endemicity of NTDS in Ga West. *Source: Ghana-Coendemicity Status* https://espen.afro.who.int/countries/ghana

The Ga West Municipality is currently one of the 29 Metropolitan, Municipal and District Assemblies (MMDAs) in the Greater Accra Region [14]. The population according to the 2010 census was 217,091 with a growth rate of 3.4%. The population is mainly concentrated along the peri-urban areas of the municipality particularly on the border with the Accra Metropolis and Ga East District [15] The municipality has the co-endemicity of lymphatic filariasis, soil transmitted helminthiasis and schistosomiasis [16]. Refer to Fig. 1 below.

**Fig. 2:**
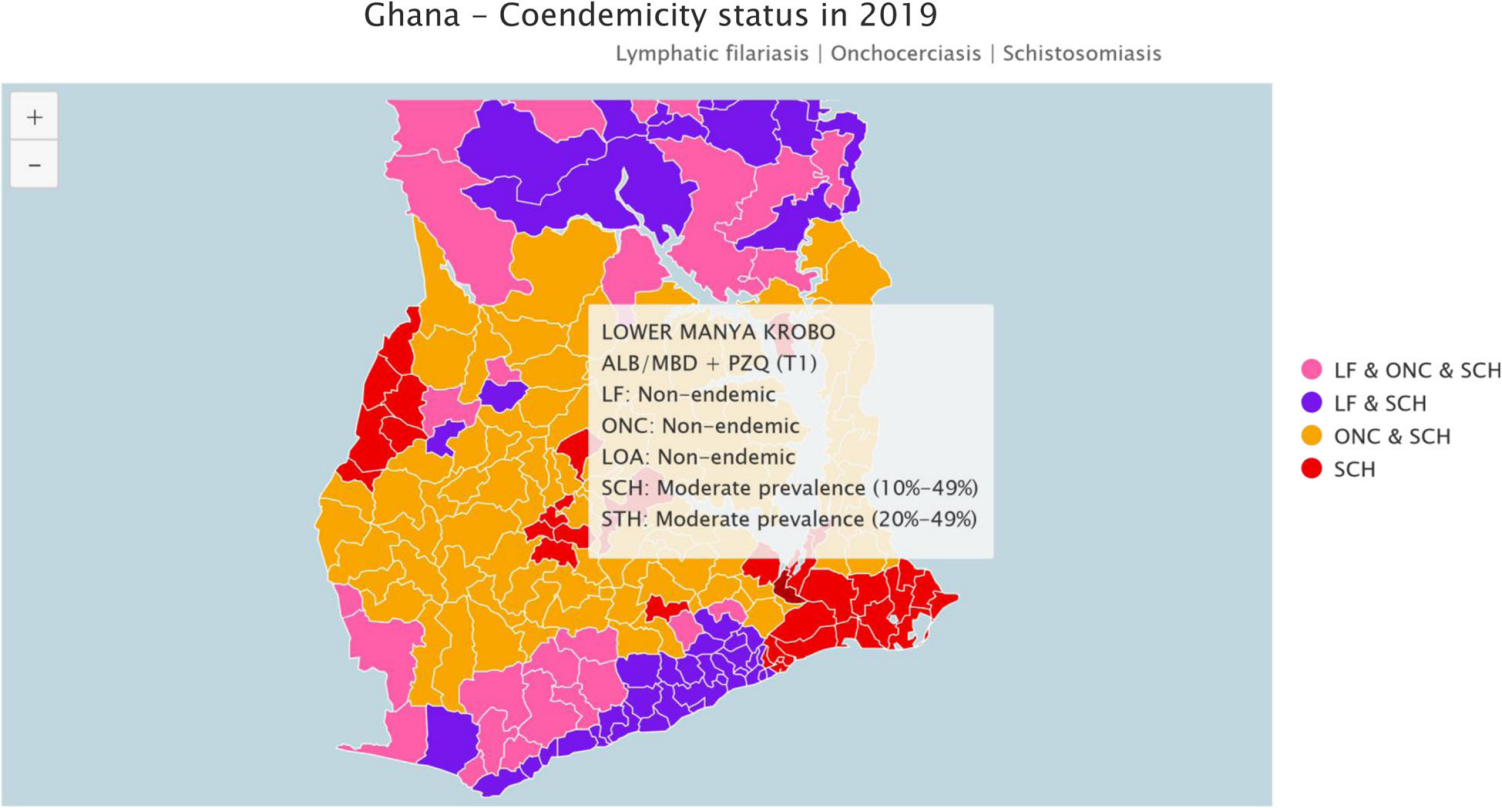
Map showing endemicity of NTDS in Lower Manya Krobo. *Source: Ghana-Coendemicity Status* https://espen.afro.who.int/countries/ghana

The Lower Manya Krobo Municipality is one of the 32 Metropolitan, Municipal and District Assemblies (MMDAs) in the Eastern Region of Ghana. The municipality covers an area of 304.4 km^2^, with a population density of 293.2 persons per square kilometer [15]. The 2010 population census estimates the population of Lower Manya Krobo Municipality to be 89,246 which is 3.4% of the total population of the Eastern Region [15]. The municipality has the co-endemicity of soil transmitted helminthiasis and schistosomiasis [16]. Refer to Fig. 2.

### Study population

Population consists of programme officers, disease control officers and other staff of the Ghana Health Service at the national, regional and district levels who play various roles in the implementation of the neglected tropical diseases programme in Ghana.

All programme officers, focal persons and staff directly responsible for the implementation of the NTD programme of the Ghana Health Service national, regional and district levels were included. All other programme officers who do not carry out any direct activities of implementation of the programme were excluded.

### Sampling

A combination of purposive and snowballing techniques (non-probability) was employed to recruit 18  participants at the three levels of the Ghana Health Service who had relevant knowledge and experience in the implementation of the NTDs programme (Refer to Table 1). Heads of the NTDs programme at the three levels were purposively sampled first for participation in the study. A snowballing technique was then employed to identify all the other staff directly working with the NTDs programme.

**Table 1:** Summary of Respondents Interviewed

Organizations/Units	No of participants
National Programme Officers	6
Regional Heads of Disease Control	2
Regional NTDs Focal persons	2
District Heads of Disease Control	2
District NTDs focal persons	2
District Disease Control Officers	4
**Total**	**18**

Source: Field Data (2020)

### Data collection techniques/procedure

A semi-structured interview guide was designed to collect data from respondents of the study. The interview guide was adapted from the WHO’s methodology for evaluation of NTDs programme [17] and checked against available literature to ensure there was no ambiguity. The guide has been applied in a mid-term evaluation in Nigeria [18].

A systematic approach was used in the collection of data. Face-to-face key informant (KI) interviews were conducted by the researcher who was the interviewer and an assistant who acted as the note taker and recorder. Interviews were started after the study was explained and written informed consent taken from the individual participants. Privacy and confidentiality were ensured throughout the data collection process. Interviews were conducted in English and audio recorded. These recordings were used as references and compared to notes that were taken during the KI interview to clarify issues that were ambiguous. Recordings were then played to respondents immediately to validate the content after the interview, transcribed and typed into Microsoft word document with security password.

### Data management and analysis

Transcription was done by the researcher and assistant after which transcribed data were compared to reach a consensus on the accuracy of content. Data collected were analyzed explicitly using thematic analysis. Textual data from transcription was read through again after which coding was done and sub-themes and patterns generated with the help of NVivo 11. Transcripts were checked multiple times by the researchers and assistants for errors. Study objective was used as primary theme and sub-themes emerged from the data as coded (Refer to Table 2). All sub-themes were from exploring the patterns and relationships within the interview data generated and checked manually to ensure its meaningfulness to the study with discrepancies resolved (Table 2).

**Table 2:** List of sub-themes from data

Theme	Sub-themes
Implementation success determinants	Donor support Education and training Management commitment and support Reliable health structure Integrative nature of programme Clear roadmap Programme resources Partnerships and collaborations

### Findings

All the 18 participants were engaged on the determinants of a successful NTDs programme implementation. The results discussed under the determinants were the most common themes that emerged from the discussions irrespective of the level of engagement across the programme.

### Determinants of implementation success

Findings from the study indicate that factors that determine implementation success of the NTDs programme are donor support, community acceptance, education and training, management commitment, reliable health structure, integrative nature of the programme itself, clear roadmap on NTDs, programme resources and partnerships and inter-sector collaboration.

### Donor support

Respondents at all levels acknowledged the immense contribution of donors as a major factor driving the success of implementation of the NTDs programme. It was noted that all programme support in terms of financial resources and intervention drugs are fully sponsored by external/international donors. Also, most of the technical and field work in relation to NTDs are solely sponsored by donors/agencies.

*“We cannot survive without our external donors as they are the main supporters of the programme; it is their money and they ensure we have the resources”.*

National programme officer 3.

*“I will say one thing that facilitates implementation is the financial support we get from the external people. Without them the programme will not stand”.*

National programme officer 1.

These donors make available the needed funds to ensure implementation and to a large extent sustainability of the programme. The donor agencies/partners referred to by respondents were identified to include foreign governments, non-governmental organizations, world health agencies, bi-national co-operations and agencies, civil society groups for NTDs and development co-operations.

*“The efforts being put in by the donors and partners. I mean the foreign agencies, NGOs and also UN and WHO. It is what is making a head way in terms of funds and medicines to run the programme”.*

National programme officer 6.

Despite majority and the main donors being external partners, respondents also noted that, donors at the district levels sometimes include local agencies who have interests in NTDs and also whose work affects communities where NTDs are endemic.

*“over here, we also have some local people. Like Volta River Authority, they contribute to Schistosomiasis control. Well, this is because of the Volta river and the communities around it”.*

Regional Disease Control Officer 3.

Respondents were unanimous on the benefits of donor support and noted that these support in terms of financial and technical resources were the main drivers of the programme from the national to the district level.

### Community acceptance

Community acceptance and mobilization was identified by respondents to be one of the major determinants. It was indicated that since the commencement of the programme, the success of community-based activities, mass drug administration and sustainability have heavily depended on the acceptance and commitment of community members residing in the programme areas and their leaders.

*“So, if you say take the medication and they say we won’t take it, what else can you do. So, the community accepting and knowing that we have such a challenge and these guys have just come to support us, they owning the programme and leading the way always made the work easy”.*

National Programme Officer 5.

The level of acceptance of a particular community where diseases exist and requires a concerted effort for control has been a great tool used to advance success in the implementation of the NTDs programme. Respondents added that, acceptance of the programme increases the understanding of beneficiaries in relation to signs and symptoms, diagnostic investigations, preventive measures, case management and impact on individuals and the economy.

*“It is about the people’s acceptability. The willingness of the people at the district and communities to help support. So far their small support is what we are sitting on”.*

District NTDs focal person 2.

On how officers ensured acceptance of the programme in the communities, it was reported that, engagement of the local authorities and their understanding created room for adequate planning and tailoring of activities to the context of the people considering their social determinants of health and needs. It also empowered community members and leaders to build capacity and reorient decision making and implementation towards a bottom-up approach.

*“we go to the chiefs and opinion leaders. You see, they are the ones the people so trust so when they talk to them that these people are coming, they understand and make our work easy”.*

District Head of Disease Control 2.

To support this, an account was shared of a programme team being chased out of a community due to improper community entry, poor information flow and lack of leadership engagement. This was to buttress how vital community acceptance has been to the programme.

*“I remember a team was going to take samples for onchocerciasis at dawn. They had to be chased out because the people did not know. They were accused of taking blood for rituals. It is like that”.*

Regional NTDs focal person 1.

### Education and training

Findings from respondents at all levels showed that planned and periodic education of beneficiary communities on NTD interventions served as an important determinant of programme success. Due to the vast knowledge gap and lack of awareness of community members on the diseases classified as neglected, constant education helps them understand the causes of diseases, its preventive measures and also helps communities especially affected communities appreciate the impact and burden of these diseases.

“*… and organizing training for the communities is one of the best strategies. At least it makes them understand what we are doing”.*

District NTDs focal person 2.

The health education also ensures that people are equipped with the requisite knowledge on signs and symptoms of various neglected tropical diseases and preventive measures. Some respondents explained that, the education helps in easy case detections and enhancing reporting of changes including complications. In some circumstances, health education is complimented with counselling sessions. Almost all respondents reported that, training of programme officers, community volunteers and other stakeholders involved has been very instrumental in ensuring effective implementation. When officers are constantly trained on the programme strategies as well as contemporary methods of managing health programmes, it equips them with adequate knowledge and skills on the most appropriate ways of implementation. Also, the training ensures that officers are updated on evidence-based practices as well as learning from other countries who have made major impact.

*“One thing that has helped us are the trainings we do across the country. We hold different trainings for our staff and volunteers. It is like refresher training and it has been very beneficial”.*

National programme officer 5.

It was further noted that, training of programme officers is carried out differently at the various levels of the health system in a cascaded form. Thus, national officers are usually trained by external partners and consultants. Regional level trainings are organized and facilitated by the national programme where knowledge gained from national training is passed on using various methods of teaching and learning. Similarly, focal persons at the district levels are then trained by regional officers on a rolling basis gradually to the community health workers and volunteers.

*“We do a cascaded training from the national TOT to the regional TOT to district and volunteers training”.*

National programme officer 1.

Also, to stress the point of education and training at the district level, respondents mentioned that training of teachers who also serve as volunteers had a positive impact under the school health programme and had been one of their success stories.

*“Right now, hmmm it is our teachers in the school that have made the programme work. And we train them on what they have to do.*

District NTDs focal person 2.

### Management commitment and support

Respondents reported that the support of management of the national programme, regional and district health directorates have also contributed positively to ensuring effective implementation. They noted that with the structure of the programme, it would have been impossible to implement if management do not show maximum support. Management actions such as prompt decision making, timely signals to sponsors, procurement of necessary logistics and support for the mission and vision of the programme were mentioned. The commitment of leadership to positively influence public health at the local level has played a major role in continuous advocacy and support towards specific objectives.

*“When it comes to people, I can only say our management are the ones making things work. It is because they are interested in the programme’s mission and vision”.*

Regional NTD focal person 2.

The constant communication among leadership, administrative support, sharing of knowledge, innovation and vision of the management has proved to increase the chances of meeting the programme objectives. Respondents further clarified that their classification of management does not include political figureheads.

*‘Sometimes with the decisions our directors in the office take you can see they really want the thing to work. They do everything to make sure things are implemented well”.*

District Disease Control Officer 3.

*“Management, I don’t mean the political people, I mean the programme manager and other consultants. They are part of the reasons why implementation is effective”.*

National Programme Officer 2.

Also, it was captured that the efforts of management to identify gaps and challenges in intervention implementation itself are a major boost to all officers and motivation for all implementing bodies. They also form action teams and working units to respond to peculiar situations. Respondents noted that the support goes a long way to ensuring that every officer contributes more and has been one of the major driving forces for meeting goals and objectives.

### Reliable health structure

The governance and service delivery structure of the public health sector in Ghana was reported as one of the key elements ensuring effective implementation of the NTDs programme. Respondents reported that organizational structure of the Ghana Health Service which is the state agency under the Ministry of Health responsible for implementation of the NTDs programme has been of immense benefit to the programme. This theme covered both leadership and service delivery structure.

*“… one of the factors I must say is that we have a reliable health structure in the country. It is the two. leadership and service delivery”.*

National Programme Officer 5.

The main organizational levels of healthcare mentioned were the national, regional, district and sub-district levels. Respondents noted that the degree of autonomy and oversight given to the above levels ensures that there is adequate decision making and communication through the health structure. It was noted that this structural differentiation birthed the public health division which has special oversight over public health and disease control programmes in the country. This ensured that adequate attention is given to specific vertical and non-vertical public health interventions such as the NTDs programme under the disease control unit.

*“You see the way we work in Ghana Health Service, there are structures in place for everything. I think that is one thing that has made us achieve most of our success. We just follow the structures”.*

National Programme officer 1.

In order to juxtapose the importance with the mass drug administration, a respondent gave a description that the NTDs programme does not recruit any new people for work. It sends adequate instructions through the health system structure to all regional directorates who further inform their districts of the intended programmes. This ensures preparation for the national teams and also ensures that disease control officers who are familiar with the local settings are engaged to make it effective.

*“We don’t just get up and go. Like, we are going to the northern region. The national informs the region for them to also notify the district through letters. A team will be coming to do ABCD kindly give them your support. That is what works”.*

National Programme officer 4.

### Integrative nature of the Programme

Upon further analysis of data from the interviews, it was clear from respondents that the integrative nature of the NTDs programme in the country has been a contributing factor to the successes achieved. It was captured that the harmonisation of diseases classified as “neglected” under one umbrella body with same targeted efforts has ensured cost-effective and more efficient ways of achieving the objectives of control and elimination using little resources.

*“At first a lot of the diseases were having their own programmes. But now we have a lot under one programme and it is helping since same resources are used to achieve same purpose”.*

National Programme Officer 4.

It was stated that the programme by its nature targets multiple diseases through joint similar approaches. In addition to these, some respondents mentioned that the integration has brought the opportunity of having a clear and readily available data obtained from the integrated diseases surveillance and report sheets available at all levels and entered into the District Health Information Management System (DHIMS). This was mentioned as a system that reported on all diseases within the health system which enhances understanding and appreciation of the burden of disease.

*“The level of integration of the programme into the health system is now high. So now we enter our data into the DHIMS. It is there for everyone to access. So now decision making is easier”.*

District Head of Disease Control 1.

Respondents added that the integration of activities within the programme into the main stream health services structure has been beneficial to ensuring case identification and management. The engagement of various health facilities from the CHPS level to tertiary levels have ensured that neglected tropical diseases are being identified and managed at all levels of healthcare. It also ensures that more severe cases are effectively referred through the referral system. Respondents mentioned that, some hospitals have specially trained officers in charge of NTDs who compliment the activities of various disease control officers and NTDs control focal persons in the districts.

*“In the district hospital, we have some health workers who pay special attention to managing some NTDs cases. So, they also help in clinical surveillance”.*

District Disease Control Officer 4.

*“You see at first we let them come to the health directorate when identified. But now even the CHPS centres have been empowered to give programme drugs. You have to go through your community facilities first”.*

District NTDs focal person 2.

### Clear roadmap

The role of a clear roadmap and a plan for the NTDs programme came out as an essential factor for implementation success and was stressed more at the national level. Respondents noted that the existence of a clear policy guideline and strategic plan from the international level led by the WHO through to the country level has greatly directed the actions of the programme and helped in implementation. They noted that it clearly spells out the objectives of the NTDs programmes, and methods for achieving such objectives.

*“Our work is guided by some guidelines that have decided all what we have been doing. WHO has a whole document on NTDs so our public health division also follows that and adapt it to work for the country”.*

National Programme Officer 3.

It was further noted from the interviews and documents analysis that, the NTDs masterplan expressly provides a country specific situational analysis and defined disease hotspots for all NTDs in Ghana with implementation plans and strategies.

It also outlines and defines the specific roles and responsibilities of all stakeholders and spells out the strategies for integrations, collaboration and partnership. Participants at the national level added that, in deciding upon yearly goals and intra-programme activities, management draws in-depth information from the masterplan to guide decision making. In effect, it was captured that, the presence of this roadmap has ensured a sustained effort towards prevention, control and elimination of the NTDs in Ghana. A thorough analysis of the Masterplan for Neglected Tropical Diseases Programme Ghana (2016–2020) revealed that, the document provides strategic direction for the NTDs programme based on financial and geographical accessibility, quality of care, partnerships and equitable distribution of resources and efficiency.

The document was widely available at the national level. Regional and district officers were aware of the masterplan and how it controls activities of the programme but do not have copies available. They also do not select what specific activities should be implemented at a particular time since that was solely the responsibility of the national programme. However, it served as a reference document for all from time to time.

*“The plan is there; we also draw our yearly plan for NTDs based on what the national sends to us. Sometimes we even draw ours and await theirs. It has helped us plan well”.*

Regional head of Diseases Control 1.

*“We have all seen the masterplan but you can’t get one now. We draw our own too because it is not all the diseases we have here. We target just the ones here”.*

District NTDs focal person 1.

### Programme resources

In the absence of some number of resources, it was noted that it will be impossible for the programme to meet its key deliverables. The availability and timeliness of resources was reported at all the levels of implementation as a major facilitator of implementation success.

*“Even though we do not get all that we want, I think the funding we are getting now has played a major role. Else we won’t be doing anything”.*

National programme Officer 2.

In breaking down the resources instrumental to implementation so far, financial resource was the most mentioned among all respondents but also noted were capital and human resources. Respondents at the national level reported that financing of the programme was mainly from external partners and non-governmental organizations dedicated to the control of NTDs. Their provision of funds to the programme has ensured that the programme is able to meet the costs associated with various processes. This involves procurement of drugs for mass drug administration which is a major activity in the control of diseases. The availability of financial resources has also facilitated the remuneration of community volunteers to motivate them for continuous work.

*“All activities we have been doing is when they bring the funds. So far, they have been doing well on bringing some money and that one is a big push for us”.*

Regional NTDs focal person 2.

Respondents explained that adequately skilled officers have been the back bone for effective delivery. The existence of well-trained public health officers and clinical officers such as medical doctors and laboratory scientists on the programme ensures that all aspects of health are covered. Also mentioned are the well-trained community volunteers who were responsible for community outreaches and home services.

*“… even the officers here are one of the reasons things are working. At first officers were not assigned to only NTDs but now our human resource is okay”.*

National Programme Officer 4.

### Partnership/inter-sector collaboration

Findings from the interviews identified partnerships and collaborations among various sectors in the country as a factor that ensures continuous and effective implementation of the NTDs programme across the country. It was established that programme managers were aware of factors such as economic situation, social environment, education, politics, income and social position that greatly influences the health and uptake of services at the individual and community level. The collaborations were in the forms of forming committees with representation from all identified sectors within and outside the health setting.

*“The links we have with some other sectors are good. We partner with some of them because we know that politics, position and other things are important especially when doing Mass Drug Administration”.*

National Programme Officer 4.

Additionally, respondents noted that in order to adequately reduce the burden of the NTDs, it was necessary to engage other stakeholders because health was not being driven by the health sector alone. The programme’s collaboration with other sectors such as education, water and sanitation, Metropolitan, Municipal and District Assemblies, state agencies, other vertical disease control programmes and community leaders ensure that there is sustained awareness and also co-benefits as a result of their duties played individually and together.

*“Our partners in this region help us make things easy and get the programme in line. The Education Ministry and Assemblies help us a lot especially with our school health programme”.*

Regional Head of Disease Control 1.

These collaborations were present across all national, regional and district levels of implementation although more collaborations were reported at the national level as compared to the lower levels. However, each level developed some form of engagement with particular groups for mutual benefit. Some benefits reported by respondents include enabling officers and volunteers have easy access to target groups, ensuring that adequate resources and information relating to the sanitary causes of diseases were addressed, poverty which was a central issue of NTDs tackled, issues of stigmatization dispelled by community authorities among many others.

One of the major examples shared by respondents at the district level was the programme’s partnership with the School Health Education Programme (SHEP) under the Ghana Education Service. This programme was in place to spearhead implementation of beneficial health-educational programmes in the educational sector of Ghana. The NTDs programme collaborates with this unit to carry out its school health and Mass Drug Administration (MDA) campaign. Respondents noted that, the existence of this partnership makes it easy for obtaining necessary clearance, conducting training and administration of programme drugs to school going children.

Secondly, the collaboration with the Ghana School Feeding Programme (GSFP) has ensured that school children are provided with adequate meals to enhance their nutritional status since these NTDs have negative impacts on the nutrition and intellectual capacity of these children. It also ensures that food is made available for children during drug administration periods to prevent side effects from the strength of the drugs.

*“Over here, the SHEP coordinators make things easy for us. Also, they are under the education ministry, and you know that is government. So, they make things easy for us to do”.*

District NTDs focal person 1.

Other collaborations were with the Ministry of Sanitation and Water Resources and the MMDAs to provide adequate sanitary conditions and safe water to communities in order to tackle water related NTDs such as Lymphatic Filariasis, Soil Transmitted Helminths and Onchocerciasis. This is in line with the endorsement of the WHO that access to safe WASH services are instrumental to the prevention, intensified control, management and elimination of NTDs.

A striking partnership reported by a district officer was one with the traditional and spiritual healers in the communities. This was in place due to the knowledge of the health seeking behaviour of community members. Most of the people with these conditions resort to traditional and spiritual healers as the first contact for cure due to the myths of spirituality as the cause of diseases. Hence this collaboration made it possible for such people to be reported to the various district officers when identified by the traditional healers. However, this partnership though existent has its own challenges.

In addition to these, respondents added the support of local stakeholders such as the community leaders, Volta River Authority, heads of health facilities as forms of collaborations. They ensure that, implementation is smoothly carried out in the respective constituents in order to meet programme objectives.

*“It’s the collaboration with Ghana Education Service, we don’t do things in isolation. We work with them because they make it easy when schools and drugs are involved”.*

Regional Head of Disease Control 2.

*“The traditional healers in the community are part. We go to them so that they notify us when they get those kinds of cases”.*

 District Disease Control Officer 3.

## Discussion

Findings on the determinants of successful implementation revealed that support from partner and donor agencies, education and training, partnerships and inter-sectoral collaboration, the structure of the health system, resources available for programme activities, the existence of a clear roadmap for NTDs control and elimination, community acceptance and support form management of the programme were major factors that ensured effective implementation. Similar findings in this study were reported in a systematic review conducted on the factors that ensure effective implementation of mass drug administration in Sub-Saharan Africa [19]. This study reported awareness creation through health education, partnerships and collaborations, integration of the MDA with already existent health programmes and provision of incentives and training as the major facilitators. Other factors which were reported were the motivation of community distributors, morbidity management programmes and management of adverse effects [19].

A similar scoping review on the implementation of health promotion policies reported a collaborative and effective local planning and action, effective leadership availability of resources and knowledgeable staff and motivation of community drug distributors [20].

Training, availability of resources and effective collaboration were reported in both original researches [21, 22] and systematic reviews [23] across health policies and programmes implementation.

A study of national clinical programmes in Ireland though showing different contextual features as compared to Ghana, reported leadership as the biggest facilitating factor which was in line with management commitment and support in this study. The former stressed that availability of resources was important but was not within the control of the national clinical programme [24]. This study employed qualitative means of enquiry however, a different study using quantitative methods also reported readiness for implementation, organizational culture and implementation climate which though different from findings in this study were within similar constructs [25]. Complimenting these findings, a mixed method study on the implementation of community-based health interventions in Argentina also stressed that inter-sectoral participation as seen in this study was key to implementation success in addition to motivation of community leaders and provision of local resources [26].

One major facilitator revealed in this study was the support of donors and partners. Similar theme was reported in a qualitative study on the perspectives of integrated neglected tropical diseases programme. This study noted that the influence of funders was very essential and critical to ensuring the effectiveness of NTD programmes [27]. Considering a study in a fellow Sub-Saharan country and out of the health domain, findings from the determinants of successful project implementation in Nigeria revealed top management support, client acceptance and human resources which were similar to those reported in this study [28].

Findings from the study also corroborated with other qualitative studies in South Africa on HIV programme. From these studies, standardized training and existence of good organizational structure were noted which are similar to training and education and reliable health structure reported in this study [29, 30].

The importance of leadership support was highlighted in a study out of the health domain. The study considered the factors that determine effective management of public financial management policies and systems. Quantitative results indicated that leadership has a significant effect on effective management of financial management policies especially in the public sector [31].

An evaluation of all these studies in relation to findings of this study shows that despite the policy, programme or sector, several factors are similar and cut across. However, some factors that facilitate implementation are context specific and are in relation to the activities of a particular programme.

### Limitations

The study considered the state implementing organization. Other non-state implementing organizations exist and could provide other perspectives to implementation of the programme.

The study employed qualitative methods of inquiry. However a quantitative or mixed method could reveal other important factors.

## Conclusion

Though the NTDs programme faces challenges, it strongly draws on factors such as the support from donors, partnerships and community acceptance from within its outer setting for success. Also, the commitment of management, education and training and reliable structure of the health service were inner factors that have important to programme success. It is expedient for programme managers and all stakeholders especially the government to maximize the determinants to ensure effective implementation.

### Recommendations

The study highlights the necessary factors needed to ensure the continuous implementation and sustenance of the NTDs programme. It is essential for all stakeholders to continuously develop educational and training modules for both programme officers and community members.

Sustainability plans should be developed taking into consideration yearly roadmaps, structures for stakeholder management and ways of ensuring continuous availability of programme resources.

It is also recommended that; policy makers involve future implementers and beneficiaries in developing policies and their implementation frameworks through partnerships and collaborations to ensure fluidity.

Further studies drawing on mixed and quantitative approaches considering a wider range of stakeholders are recommended in addition to studies on effectiveness of implemented interventions.

## Data Availability

Available data is freely presented in the summaries within the result section. All data and original transcripts are available with the corresponding author and will be made available upon reasonable request.

## Abbreviations

ESPEN: Expanded Special Programme for Elimination of Neglected Tropical Diseases
MDA: Mass Drug Administration
MMDAs: Metropolitan, Municipal and District Assemblies
NTDP: Neglected Tropical Diseases Programme
NTDs: Neglected Tropical Diseases
TOT: Trainer of Trainees
